# Anaesthesiological implications of Kimura's disease: a case report

**DOI:** 10.4076/1752-1947-3-7316

**Published:** 2009-06-25

**Authors:** Massimiliano Sorbello, Alessandro Laudini, Gianluigi Morello, Mirco Tindaro Sidoti, Jessica Giuseppina Maugeri, Alessia Giaquinta, Tiziano Tallarita, Daniela Corona, Domenico Zerbo, Alessandro Cappellani, Pierfrancesco Veroux, Laura Parrinello, Massimiliano Veroux

**Affiliations:** 1 Department of Surgery, Transplantation and Advanced Technologies, Anaesthesia and Intensive Care Unit, University Hospital of Catania, Via Santa Sofia, 95123 Catania, Italy; 2 Department of Surgery, Transplantation and Advanced Technologies; Vascular Surgery and Organ Transplant Unit, University Hospital of Catania, Via Santa Sofia, 95123 Catania, Italy

## Abstract

**Introduction:**

Kimura's disease is a chronic inflammatory condition belonging to the angio-lymphatic proliferative group of disorders, usually affecting young men of Asian race, but is rare in Western countries. It is a benign but locally injurious disease, of unknown aetiology, whose classical clinical features are a tumour-like swelling, usually in the head and neck, with or without satellite lymphadenopathy, often accompanied by eosinophilia and elevated serum IgE.

**Case presentation:**

We report the case of a 33-year-old Caucasian woman with an atypical localization of Kimura's disease, discussing the anaesthesiological implications and reviewing the current literature on Kimura's disease.

**Conclusions:**

The diagnosis of Kimura's disease can be difficult and misleading, and anaesthesiological precautions could be ignored. Patients with this disease are often evaluated for other disorders: unnecessary diagnostic tests and investigations, or even surgery, may be avoided by just being aware of Kimura's disease.

## Introduction

Kimura's disease (KD) is a chronic inflammatory condition belonging to the angio-lymphatic proliferative group of disorders, usually affecting young men of Asian race but is rare in western countries. It is a benign but locally injurious disease, of unknown aetiology, whose classical clinical features are a tumour-like swelling, usually in the head and neck, with or without satellite lymphadenopathy, often accompanied by eosinophilia and elevated serum IgE [1,2].

We report a patient with atypical localization of KD and, referring to a literature review, discuss the anaesthesiological implications.

## Case presentation

A 33-year-old Caucasian woman, weighing 57 kg, was admitted to our hospital for abdominal pain, haematuria and right flank tumefaction. These symptoms started two weeks earlier, three months after the end of a pregnancy. A computed tomography (CT)-scan showed a mass involving the right ureter, probably of retroperitoneal origin. Her medical history was relevant for an exploratory laparotomy three years earlier for an intra-abdominal mass; histologic examination was suggestive of an atypical localization of KD.

A relaparotomy was planned to resolve renal and ureteral compression. Anaesthesiological evaluation revealed asthma treated with beclomethasone and salmeterol inhalation, insipid diabetes with a urine output 2500 mL/day, controlled with nasal desmopressin and polyarteritis nodosa-like vasculitis. Laboratory test results showed a normal white blood cell (WBC) count with eosinophilia, and normal renal functionality parameters; urinary tests indicated no microalbuminuria and sporadic red blood cells (RBCs).

Promethazine 50 mg was given intramuscularly the night before surgery, and midazolam 2.5 mg intravenous was given as premedication, followed by morphine 10 mg intramuscularly and desmopressin endonasal spray (1 puff/side). Before anaesthesia induction, a peridural catheter was placed at the T12 level, and after aspiration test and test dose administration, ropivacaine 50 mg in 10 mL saline was given, followed by a continuous 5 mL/hour^−1^ infusion (25 mg*hr-1). Thiopental sodium (TPS) 4 mg/kg^−1^ and fentanyl 1.5 mcg/kg^−1^ were used to induce general anaesthesia, followed by cisatracurium 0.2 mg/kg^−1^ to achieve endotracheal intubation. Anaesthesia was maintained with sevoflurane 1 minimum alveolar concentration (MAC), fentanyl and cisatracurium on demand and volumetric mechanical ventilation (tidal volume (TV) = 8 mL/kg, respiratory rate (RR) = 12/minute, inspiratory/expiratory (I/E) = 33%).

The surgical procedure included neoplastic mass resection, ureteral repositioning and transvesical positioning of two ureteral stents. Fluids were administered according to a surgical procedure related fluids protocol (8 mL/kg^−1^), renal function being preserved via fenoldopam (0.1 mcg/kg^−1^/min^−1^) continuous infusion, according to our kidney transplant nephroprotective protocol [3]; urine output was 2 mL/kg^−1^ during surgery.

Anaesthesia emergence was uneventful, and no respiratory distress was noticed. The patient was then moved to a post anaesthesia care unit (PACU) for postoperative multiparametric monitoring. Postoperative nausea and vomiting (PONV) prophylaxis was performed; ropivacaine peridural continuous infusion by elastomeric pump was administered for postoperative pain, and inhalation and desmopressin treatment were confirmed during PACU recovery, with good results on pain control, respiratory function and urine output. The postoperative course was uneventful, and the patient was discharged on postoperative day 10. Histopathological examination confirmed the diagnosis of atypical abdominal location of KD. At a 6-year follow-up, the patient is doing well without signs of recurrence.

## Discussion

Kimura's disease is considered endemic in East Asians, while in non-Asian individuals, only sporadic cases have been reported. A recent retrospective review of 21 histopathology specimens conducted in the United States by the US Armed Forces Institute of Pathology [1] found the following racial distribution of KD: seven Caucasians, six African Americans, six Asians, one Hispanic, and one Arab, concluding that, though rare, if clinically suspected, KD should be included in the differential diagnosis for people of any racial group. KD is usually seen in young adults, with most patients being aged between 20 and 40 years of age [2]; men are affected by KD more commonly than women, with a 3:1 ratio [2].

The aetiology of KD is still unknown; a number of theories have been suggested for the origin of KD, including impairment or interference with immune regulation, atopic reaction to a persistent antigenic stimulus by arthropod bites, virus [2] and neoplasm. The most interesting hypothesis suggests Candida ssp acting as a source of persistent antigenaemia, although neither hyphae nor spores have been isolated [2].

The disease is manifested by an abnormal proliferation of lymphoid follicles and vascular endothelium. Peripheral eosinophilia and the presence of eosinophils in the inflammatory infiltrate suggest that KD might be a kind of hypersensitivity reaction. Several lines of evidence indicate that lymphocyte T-helper 2 (Th2) might also play a role [2].

Several clinical features characterize KD: patients may present with a solitary enlarged painless lymph node or generalized lymphadenopathy (67% to 100%) [2]. Salivary gland involvement is also frequently observed [2]. Other findings include single or multiple subcutaneous nodules, which are usually located on the head or neck, especially in the peri-auricular, parotid, or submandibular regions; these lesions, isolated or multiple, occur as deeply seated, large soft tissue masses in the subcutis or salivary glands, without significant change in the underlying skin [2]. Less frequently, the eyelids, orbit, and lachrymal glands may be involved, with an average lesion diameter of 3 cm [2].

Less common localizations of KD include the epiglottis, larynx, tympanic membrane, median nerve and spermatic cord, elbows, heart, axillary or popliteal region, and chest wall [4]. Rarely, KD may present as an intra-abdominal mass [5].

KD might also be associated with peripheral manifestations of vasculitis, typically eosinophil vasculitis; histologically, KD is characterized by florid lymphoid infiltrates with prominent lymphofollicular hyperplasia, vascularization of germinal centres, marked eosinophilia with eosinophil abscess formation and proliferation of small veins, showing mild to moderate vascular proliferation [6].

KD is often associated with autoimmune diseases such as ulcerative colitis and more frequently, similar to our case, with bronchial asthma [7], typically responding to steroids but not to treatment with theophylline or other bronchodilators [8].

Coexisting renal disease is common, with an incidence ranging from 10% to 60% [7], while 10% to 12% of patients may suffer from nephrotic syndrome [7] characterized by clinically relevant proteinuria in 12% to 16% of cases [4]. Renal impairment is probably due to immunocomplex mediated damage or to Th2-dominant immune response disorders.

The diagnosis of KD is not easy, and differential diagnosis includes inflammatory and neoplastic conditions, tuberculosis, angiolymphoid hyperplasia with eosinophilia (AHLE), cylindroma, dermatofibrosarcoma protuberans, Kaposi's sarcoma, pyogenic granuloma and other infectious lymph node enlargements for example, toxoplasmosis.

Ultrasound, CT and magnetic resonance imaging (MRI) might be diagnostic and can help staging the extent and progression of the disease as well as the lymph node involvement. Because of the rarity of KD in Western countries, both clinicians and radiologists are relatively unfamiliar with some pathognomonic findings of this disease, thus leading to unnecessary diagnostic tests and investigations [4].

The diagnostic challenge of KD is generally solved by histological study: although there is no specific diagnostic feature of Kimura's disease, fine-needle aspiration cytology is helpful in some cases, and definitive diagnosis can be obtained by histologic examination of the excised lesion [2].

The gross lesion may or may not show obvious foci of necrosis. Microscopically, the key findings are marked hyperplasia with pronounced eosinophilic infiltration. Foci of necrosis are seen with vascularization of the paracortex and deposition of hyaline material within follicles. Polykaryotic giant cells are a common feature. This combination of histologic findings is very characteristic of Kimura's disease. The polymorphous infiltrate with eosinophilia and the presence of giant cells also raise the suspicion of Hodgkin's disease. This is especially true when diagnosis is attempted by fine-needle aspiration. The absence of Reed-Sternberg cells helps distinguish Kimura's disease from Hodgkin's disease [9].

An important differential diagnosis should be made with ALHE. These two diseases have some common histological features, such as eosinophilia and vascular proliferation, but ALHE is usually seen in older patients, manifesting as multiple small dermal eruptions and only rarely with lymphadenopathy, salivary gland involvement and elevated serum IgE [4].

Treatment of KD is not strictly codified and is still controversial: surgical excision of nodules is the first-line treatment, though affected by a recurrence rate up to 25%; in any case, because of the lack of malignant transformation, radical or demolitive surgery should be avoided [2]. Systemically administered steroids, typically prednisolone, show good effects on disease progression. Due to its effects on Th2 lymphocytes, cyclosporine has been described as a possible therapy for KD [10].

Radiant therapy (localized on lesions, 26-30 Gy) has also been considered in the case of steroid-resistant lesions, but its role, accounting for unavoidable side effects including secondary malignancies, should be counterbalanced by adequate benefits, especially if considering the absence of documented malignant transformation of KD [11].

This report is a singular manifestation of KD: to our knowledge, only one case of abdominal localization of KD has been reported [5]; moreover, our patient presented the typical association with eosinophilia, asthma and vasculitis, while representing the only case of association with insipid diabetes. Finally, our patient presented with a KD relapse just after the end of pregnancy.

The clinical manifestations reported in our patient contraindicated aggressive medical treatment with steroids or other medications, the hydronephrosis being rapidly progressive and requiring urgent surgical treatment (renal function was still normal on laboratory tests).

From an anaesthesiological point of view, KD offers several interesting implications, the first of which is being aware of KD diagnosis itself. To the best of our knowledge, only a single case report has been published on the anaesthesiological implications of KD [12].

In our patient, there was no cervical manifestation of KD, which may potentially grow rapidly with possible important implications on airway patency [2].

Epiglottic localization [13] could present a life-threatening situation, while being responsible for a possible "cannot ventilate - cannot intubate" scenario as soon as airway muscular tone is suppressed by hypnotics and/or muscle relaxants, with total compromise of airway patency and ventilability, and with the sole alternative of rapid tracheal access.

Similar problems might derive from laryngeal localization [14]; in both cases, extubation problems might occur so that a protected extubation should be planned, especially in the case of co-existing predicted or unpredicted difficult intubation. Laryngeal localization may also account for sleep apnoea syndrome manifestations [14]. In all of these cases, steroid pretreatment or premedication could be beneficial to reduce swelling or oedema. Awake fibreoptic intubation could be the only possible alternative in the case of large neck masses.

In the case of large but superficial masses, general anaesthesia rather than local or loco-regional anaesthesia could be preferable, whenever difficult intubation is expected, especially if excisional surgery has to be performed in the neck.

Asthma is often associated with KD, and bronchodilators, inhalation therapy and especially steroids should be administered in the perioperative course [7]. Local anaesthesia of the vocal cords or adequate analgesic administration should be taken in to account during intubation manoeuvres in order to reduce airway reflexes. Good postoperative pain control should be achieved, preferably avoiding non steroidal anti inflammatory drugs (NSAIDs), both for renal protection and as possible allergic and asthmatic triggers [3].

Whenever possible, "low stress procedures" should be recommended, with controlled anaesthesia and surgery for both invasiveness and duration [15].

Deep venous thrombosis prophylaxis, with low molecular weight heparin and early mobilization of the patient should be provided to all patients with co-existing vasculitis.

KD is often associated with various entities of renal impairment, so adequate nephroprotective strategies should be undertaken; this becomes mandatory in the case of clear nephritic syndrome with proteinuria. Careful study of renal function should be performed before surgery in the case of elective procedures; adequate perioperative hydration and perfusion should be granted, and potentially nephrotoxic drugs should be avoided [3], with particular reference, in our protocols, to contrast medium, aminoglycosides and NSAIDs.

In selected cases, fenoldopam infusion at 0.1 mcg/kg^−1^/min^−1^ could be indicated, providing nephroprotection without the effects on arterial blood pressure [3].

## Conclusion

We present an atypical manifestation of Kimura's disease occurring in a young Caucasian woman, and discuss the anaesthesiological implications on the basis of a literature review.

The diagnosis of KD can be difficult and misleading, and anaesthesiological precautions could be ignored. Patients with this disease are often evaluated for other disorders: unnecessary diagnostic tests and investigations, or even surgery, may be avoided by just being aware of KD.

## Abbreviations

AHLE: angiolymphoid hyperplasia with eosinophilia; CT: computed tomography; I/E: inspiratory/expiratory; KD: Kimura's disease; MAC: minimum alveolar concentration; MRI: magnetic resonance imaging; NSAIDs: non steroidal anti inflammatory drugs; PACU: post anaesthesia care unit; PONV: postoperative nausea and vomiting; RBC: red blood cells; RR: respiratory rate; TPS: thiopental sodium; TV: title volume; WBC: white blood cells.

## Consent

Written informed consent was obtained from the patient for publication of this case report. A copy of the written consent is available for review by the Editor-in-Chief of this journal.

## Competing interests

The authors declare that they have no competing interests.

## Authors' contributions

MS performed the anaesthesiological protocol, was a major contributor in writing the manuscript and gave final approval to the manuscript; AP interpreted the intra-operative parameters during the surgical procedure; GM performed the anaesthesiological protocol and analysed and interpreted the data regarding the postoperative course; MTS was responsible for the postoperative management of the patient; JGM interpreted and adapted the patient's postoperative therapy; AG and TT interpreted the data regarding the follow-up of the patient; DC, DZ and PV interpreted the laboratory data; AC performed the surgical procedure; MV performed the surgical procedure, was a major contributor in writing the manuscript and gave final approval to the manuscript. All of the authors read and approved the final manuscript.
